# Erythropoietin improves neurobehavior by reducing dopaminergic neuron loss in a 6-hydroxydopamine-induced rat model

**DOI:** 10.3892/ijmm.2014.1810

**Published:** 2014-06-17

**Authors:** CHEN QI, MINGXIN XU, JING GAN, XINXIN YANG, NA WU, LU SONG, WEIEN YUAN, ZHENGUO LIU

**Affiliations:** 1 Department of Neurology, Xinhua Hospital Affiliated to Shanghai Jiao Tong University School of Medicine, Shanghai 200092, P.R. China; 2 School of Pharmacy, Shanghai Jiao Tong University, Shanghai 200240, P.R. China

**Keywords:** erythropoietin, Parkinson’s disease, tyrosine hydroxylase, mitogen-activated protein kinases

## Abstract

The purpose of this study was to determine the effectiveness of the systemic administration of high dose erythropoietin (EPO) in a 6-hydroxydopamine (6-OHDA)- induced rat model. Rats were divided into 7 groups. Groups 1–4 were administered daily EPO doses of 0; 2,500; 5,000 and 10,000 U/kg via intraperitoneal injection (i.p.) for 5 days. The EPO concentration in cerebrospinal fluid (CSF) was determined by enzyme-linked immunosorbent assay (ELISA) and western blot analysis. The dose of 10,000 U/kg was then selected for subsequent experiments. In group 5, rats received saline via medial forebrain bundle (MFB). In group 6, rats received 6-OHDA via MFB. In group 7, an EPO concentration of 10,000 U/kg was constantly administered i.p. for 5 days to rats prior to 6-OHDA injection via MFB. Behavioral analysis was performed for groups 5–7 by rat rotation tests. The number of tyrosine hydroxylase (TH)-immunopositive cells in the substantia nigra (SN) was measured by immunocytochemistry. The activation of c-Jun N-terminal kinase (JNK), extracellular signal-regulated kinase (ERK), p38 mitogen-activated protein kinases (MAPKs) and caspase-3 signaling in rats were analyzed using western blotting. The results showed that there was a significant increase in EPO levels in the CSF in 10,000 U/kg group compared with the 2,500 and 5,000 U/kg groups (P<0.01). Significantly fewer rotational counts were obtained in rats that were pretreated with EPO compared with saline-pretreated 6-OHDA-lesioned rats (P<0.001). The dopaminergic neurons in the 6-OHDA-lesioned SN were also increased in the EPO-pretreated rats when compared with control rats (P<0.01). Western blot analysis revealed that EPO inhibited the 6-OHDA-induced activation of JNK, ERK, p38 MAPK and caspase-3 signaling in the rat model. In conclusion, systemic administration of a high dose of EPO exerted neuroprotective effects in reversing behavioral deficits associated with Parkinson’s disease and prevented loss of the dopaminergic neurons through the MAPK pathway.

## Introduction

Parkinson’s disease (PD) is a progressive neurodegenerative disease with clinical symptoms such as tremor, rigidity and bradykinesia and abnormal postural reflexes (1). The primary pathology of the disease is degeneration of the nigrostriatal system, which results in the loss of dopaminergic neurons and depletion of striatal dopamine. As the disease progresses, a variety of non-motor symptoms emerge due to the loss of non-dopaminergic pathways (2). The most effective therapy is replacement of dopamine with levodopa or dopamine receptor agonists. Despite their benefits, chronic treatment using these agents is associated with adverse effects including on-off fluctuations, wearing-off phenomena, or drug-induced dyskinesia (3). Therefore, it is important to develop new therapies.

The pathogenic features identified as being instrumental in the dopaminergic cell death process that occurs in PD, including oxidative stress, mitochondrial dysfunction, inflammation and apoptosis, provide targets for the development of new neuroprotective compounds (4). Erythropoietin (EPO) is a well-known hematopoietic hormone produced in the fetal liver and adult kidney. Neuroprotective effects of EPO have been previously demonstrated using preclinical models of central nervous system (CNS) diseases including focal and global ischemia, neurotrauma, autoimmune encephalitis, kainate-induced seizures, subarachnoid hemorrhage and spinal cord injury. It has been demonstrated that EPO can provide neuroprotection of neurons against experimental lesions (5). However, the exact mechanisms involved in EPO neuroprotection remain to be determined. EPO protein and receptors are detected in brain neurons, astrocytes, oligodendrocytes, microglia and cerebral endothelial cells (6). The mechanisms of EPO-induced neuroprotection include the prevention of glutamate-induced toxicity, inhibition of apoptosis, anti-inflammatory effects, antioxidant effects, and stimulation of angiogenesis. As EPO can cross the blood-brain barrier (BBB) (7), it has been reported that systemic administration of EPO may reduce neuron damage in animal models of ischemic stroke (8), traumatic brain injury (9), and spinal cord injury (10). Neuroprotective effects of EPO infusion into the striatum were clarified in a previous study (11). The typical PD rat model is the 6-hydroxydopamine (6-OHDA) model, which is currently the most commonly used procedure for obtaining an experimental nigrostriatal lesion in the animal. Recent studies (11) have shown that in 6-OHDA-lesioned rats, the abnormal activation of c-Jun N-terminal kinases (JNK), extracellular signal-regulated kinase (ERK), and the p38 mitogen-activated protein kinase (MAPK) pathway has been identified. Additionally, EPO has been shown to prevent staurosporine-induced apoptosis via STAT5, AKT and MAPK signaling pathways (39). The aim of the present study was to clarify whether different doses of EPO exert neuroprotective and neurogeneic effects on 6-OHDA-treated dopaminergic neurons via the MAPK pathway *in vivo*.

## Materials and methods

### Animals

Adult female Sprague-Dawley rats, weighing 180–220 g, were used in this study. Protocols involving the animals were approved by the Institutional Review Board of Xinhua Hospital and were performed according to the guidelines of the National Institutes of Health for the Care and Use of Laboratory Animals (NIH publication no. 80-23). The rats were housed in standard Plexiglas cages with a maximum of 5 animals per cage and had free access to food and water. Environmental conditions were strictly controlled, with a 12-h light/dark cycle, temperature of 22°C and humidity of 44%. The number of animals used was the minimum required for statistical analysis (n=98).

The rats were divided into 7 groups. In group 1 (n=14, sham), rats were intraperitoneally injected (i.p.) saline daily for 5 days. In groups 2 (n=14), 3 (n=14) and 4 (n=14), the rats were i.p. injected doses of 2,500; 5,000 and 10,000 U/kg EPO daily for 5 days. In group 5 (n=14), rats received saline via medial forebrain bundle (MFB), while in group 6 (n=14), rats received 6-OHDA via MFB. In group 7 (n=14), EPO (10,000 U/kg) was continuously administered i.p. for 5 days to rats prior to administration of 6-OHDA injection.

### EPO injections

For the systemic administration of groups 2–4, EPO was dissolved in saline, and i.p. injected at doses of 2,500; 5,000 and 10,000 U/kg on a daily basis for 5 days. For group 7, EPO (10,000 U/kg) was i.p. injected daily for 5 days prior to injection of 6-OHDA.

### 6-OHDA lesion

Rats were anaesthetized with ketamine (100 mg/kg, i.p.) prior to surgery and placed on a stereotaxic frame (SR-9M, Stereotaxic Instrument; Narishige Scientific Instrument Lab, Tokyo, Japan). The skull was exposed and a burr hole was drilled to introduce a syringe for injection of 6-OHDA (Sigma, St. Louis, MO, USA) solution containing 4.0 μg 6-OHDA/μl in 0.9% saline with 0.02% ascorbic acid (Sigma), pH 5.0. To minimize variability due to degradation of the toxin, the 6-OHDA solution was freshly prepared, kept on ice and protected from exposure to light. Each animal received two injections of 4 μl of the solution into the right MFB with a 10 μl Hamilton Syringe at the stereotaxic coordinates (from bregma and dura) of AP −3.7 mm, ML +1.7 mm, DV −7.8 mm, and AP −4.4 mm, ML +1.2 mm, DV −7.8 mm. The tooth bar was set to −2.4 mm (12,13). A total volume of 4 μl 6-OHDA was injected at a flow rate of 1 μl/min. Following the injection, the needle was left in place for 10 min and then slowly removed. The skin was sutured and the animals were removed from the stereotaxic instrument, placed on a heating pad for 30 min and returned to their cage. In this rat model, 6-OHDA caused a progressive loss of dopaminergic neurons in the substantia nigra (SN) as it was absorbed by the neuron’s terminals in the striatum and transported to the dopaminergic neurons in the SN, leading to damage of the dopaminergic neurons (14). Rats undergoing a sham lesion procedure in which only the vehicle [0.9% saline with 0.02% ascorbic acid (Sigma), pH 5.0] for 6-OHDA was injected into the MFB seved as the controls.

### Behavioral analysis

At 21 days following surgery, the animals underwent behavioral testing. Rats were injected with apomorphine (0.25 mg/kg in 0.9% saline, i.p.) and placed in a stainless steel cylindrical bowl. Net rotations (contralateral turns minus ipsilateral turns) were counted over a 30-min period beginning 5 min after the administration of apomorhine. Assessments were carried out by an observer who was blind to the animal pretreatments (15).

### Quantitative analysis of EPO in the cerebrospinal fluid (CSF)

EPO was administered into rats at concentrations of 2,500; 5,000 and 10,000 U/kg, respectively, via i.p. injection. Three hours post-injection, the CSF samples were collected via cisternal puncture as previously described (16). Briefly, the rats were anesthetized with ketamine (100 mg/kg, i.p.), and the head of each rat was fixed at a specific forward angle. The back of the neck and base of the skull were shaved and disinfected with 70% ethyl alcohol. An incision was made in the skin over the occipital bone, the fascia was retracted and superficial muscles were dissected. When the allanto-occipital membrane was exposed, the cisterna magna was cannulated by placing a 30-gauge needle, and CSF was carefully withdrawn. CSF was ejected into a 0.5 ml Eppendorf tube and frozen at −80°C. The volume of CSF ranged between 50 and 150 μl. EPO concentrations in CSF samples were measured using an enzyme-linked immunosorbent assay (ELISA) and an immunoblotting assay. Saline-injected animals were used as controls. EPO concentrations in CSF were measured by ELISA using the Quantikine IVD EPO kit (R&D Systems, Minneapolis, MN, USA). ELISA was used to detect endogenous rat EPO and EPO. A standard curve was performed as protocol ranging from 0 to 200 mU/ml EPO. The CSF samples were diluted based on prior experience to fit the ELISA standard range. The absorbance was assessed using a Bio-Tek μQuant™ Microplate Spectrophotometer MQX200 reader (BioTek Instruments Inc, Winooski, VT, USA).

### Immunocytochemistry

Seven randomly selected rats per group were anesthetized with ketamine (100 mg/kg, i.p.) and transcardially perfused with 100 ml of 0.1 M phosphate-buffered saline (PBS, pH 7.2) followed by 200 ml of 4% paraformaldehyde at 4°C. The brains were removed, post-fixed for 4 h in 4% paraformaldehyde, embedded in paraffin, and cut into 3 μm coronal sections on a sliding rotary microtome (Leica RM2235; Leica Microsystems Nussloch GmbH, Nussloch, Germany). Seven random sets of serial SN sections of each rat were collected for immunocytochemistry.

Immunostaining was carried out in sections using a standard avidin-biotin immunocytochemical protocol as previously described (17). Endogenous peroxidase activity was quenched by incubation for 10 min in 0.1 M PBS containing 0.2% Triton X-100 with 3% hydrogen peroxide. Non-specific binding sites were blocked for 60–90 min with 5–10% of the appropriate serum in PBS containing 0.2% Triton X-100. To localize TH-immunoreactivity (TH-IR) neurons a mouse monoclonal tyrosine hydroxylase (TH) primary antibody (1:1,000; Sigma-Aldrich, St. Louis, MO, USA) was used. Polyclonal rabbit antibodies to EPO (1:200; R&D Systems) and erythropoietin receptor (EPOR) (1:50; Santa Cruz Biotechnology, Inc., Santa Cruz, CA, USA) were also used. The primary antibodies were diluted in 0.1 M PBS-TX and 1% of goat serum thereby yielding the secondary antibody. Following incubation with the primary antibody overnight at 4°C, the sections were washed and incubated with the appropriate HRP-conjugated goat polyclonal secondary antibody to mouse and rabbit IgG 1:500 (all from Abcam, Cambridge, UK) for 1–2 h at room temperature. The secondary antibodies were diluted in 0.1 M PBS and 5% BSA. After washing, the antibody staining was visualized using a DAB kit (Abcam). After developing the reaction, the stained sections were mounted, dried, dehydrated and coverslipped with neutral balsam and examined using a light microscope (Leica Biosystems Nussloch GmbH).

Double-labeling fluorescent immunohistochemistry of dopaminergic and EPOR-expressed neurons was performed as previously described (18). The sections were incubated with TH antibody (1:1,000; Sigma-Aldrich) and EPOR antibody (1:50; Santa Cruz Biotechnology, Inc.) at 4°C overnight. The primary antibodies were diluted in 0.1 M PBS-TX and 1% goat serum. These proteins were detected using a mixture of Alexa Fluor 488 and Cy3 goat polyclonal secondary antibodies to mouse and rabbit IgG 1:200 (all from Abcam) for 1.5 h at room temperature in the dark. The secondary antibodies were diluted in 0.1 M PBS and 5% BSA. The sections were mounted in fluorescent mounting medium (Abcam), coverslipped, and kept in the dark at 4°C until they were examined. The specific immunofluorescence of the Alexa 488 or Cy3 fluorophores was visualized by excitation at 488 or 550 nm, respectively. Images were captured with an Olympus AX 70 fluorescence microscope using Olympus Fluoview FV1000 LSM and Fluoview software (v 1.3).

### Morphological assessment

Quantification of the TH-positive neurons in the lesioned and intact SN of each rat was measured in 5–6 coronal sections per animal using research-grade Image-Pro Plus software (Media Cybernetics, Inc., Silver Spring, MD, USA). The borders of the areas of interest were outlined from a live image with a 4X objective and the entire area of interest was examined using a 20X objective. Only TH-positive cells with an identifiable unlabelled nucleus surrounded by TH-immunolabelled cytoplasm or those with labelled dendrites were counted. Data were expressed as the number of TH-positive neurons per mm^2^ of striatum per animal. Quantification of TH-positive fibres was carried out by optical density analysis with the aid of an Image-Pro Plus software (Media Cybernetics, Inc.) using a 20x lens. TH-positive fibre staining intensity was determined in striatal areas surrounding TH-IR neurons, through the rostrocaudal extent of the dopaminergic lesion. Similar measurements were carried out in the unlesioned striatum in randomly chosen areas. The data from the lesioned side are presented as a percentage (mean ± SE) of the values from the unlesioned SN. To estimate the number of EPO-immunopositive cells, four sections from each of the three levels (rostral, middle and caudal) of the brain were examined under a 20x lens. Images of four fields per section per hemisphere were captured and the cell optical density was measured by Image-Pro Plus software.

### Tissue preparation

Animals were sacrificed with an overdose of pentobarbital (50 mg/kg body weight, i.p.), injected intracardially with 10 ml of cold saline, and the brains immediately removed. Brains were transfered to a plastic plate cooled on ice to remove both sides of the striatums and SN. Tissues were dissected and frozen in liquid nitrogen and stored at −80°C for immunoblotting. Seven rats from each group were used for immunoblotting.

### Immunoblotting

Samples were homogenized in RIPA buffer [50 mM Tris (pH 7.5), 150 mM NaCl, 0.1% sodium dodecyl sulfate, 1 mM EDTA (ethylenediaminetetraacetic acid), 1% Nonidet P-40] containing protease inhibitors (Roche Diagnostics Corp., Indianapolis, IN, USA) and 2 mM phenylmethylsulfonyl fluoride (PMSF) by sonication. The homogenate was centrifuged at 14,000 × g for 10 min at 4°C, and the pellet containing mainly nuclei and large debris was discarded. After determining the protein concentration in supernatants using the Pierce BCA assay kit (Thermo Fisher Scientific, Rockford, IL, USA), the samples were boiled 5 min in Laemmli Sample Buffer (Bio-Rad Life Science, Hercules, CA, USA). Samples containing equivalent amounts of protein were subjected to sodium dodecylsulfate (SDS)-polyacrylamide gel electrophoresis. Proteins were electrotransferred to a 0.22 μm Immobilon PVDF membrane (Bio-Rad, Hercules, CA, USA) in a transfer buffer (25 mM Tris, 192 mM glycine, and 20% methanol) with 250 mA current for 90 min at 4°C.

Subsequent to being transferred, the membrane was blocked by incubation in blocking buffer (50 mM Tris-HCl, pH 7.5, 150 mM NaCl, 0.1% Tween-20 and 5% w/v non-fat dry milk) for 1 h at room temperature. The membranes were incubated overnight at 4°C with mouse monoclonal β-actin antibody (1:5,000), mouse monoclonal TH antibody (1:5,000; both from Sigma-Aldrich), polyclonal rabbit EPO antibody (1:500; R&D Systems), polyclonal rabbit p44/42 MAPK (ERK1/2) antibody (1:500), polyclonal rabbit Phospho-p44/42 MAPK (ERK1/2) (Thr202/Tyr204) antibody (1:500), polyclonal rabbit p38 MAPK antibody (1:500), polyclonal rabbit Phospho-p38 MAPK (Thr180/Tyr182) antibody (1:500), anti-SAPK/JNK (1:500), polyclonal rabbit Phospho-SAPK/JNK (Thr183/Tyr185) antibody (1:500), polyclonal rabbit caspase-3 antibody (1:500), and polyclonal rabbit cleaved caspase-3 antibody (1:500) (all from Cell Signaling Technology, Inc., Danvers, MA, USA). The primary antibodies were diluted in TBST (50 mM Tris-HCl, pH 7.5, 150 mM NaCl, 0.1% Tween-20) and 5% w/v BSA. The membranes were subsequently washed with TBST (50 mM Tris-HCl, pH 7.5, 150 mM NaCl, 0.1% Tween-20) and incubated with HRP-conjugated goat polyclonal secondary antibody to mouse and rabbit IgG (1:2,000; Cell Signaling Signaling Technology, Inc.) for 1 h at room temperature. The secondary antibodies were diluted in blocking buffer (50 mM Tris-HCl, pH 7.5, 150 mM NaCl, 0.1% Tween-20 and 5% w/v non-fat dry milk). Bound antibodies were visualized using the enhanced chemiluminescence detection system (Millipore, Billerica, MA, USA) and analyzed semiquantitatively using Image Lab Software (Bio-Rad).

### Statistical analysis

Data were expressed as the mean ± standard deviations of the mean. Bars in the figures indicate mean values ± standard deviations of the mean. A one-factor ANOVA with Bonferroni’s post-hoc test was used to compare behavioral rotation rates, cell counts, and optical density between each of the treatment groups. A paired Student’s t-test was used to observe differences between the lesion and unlesion side within groups. Prior to this, homogeneity of variance between the various groups was ascertained. Differences at P<0.05 were considered statistically significant. Statistical tests were performed with SPSS 17.0 software (SPSS Inc., Chicago, IL, USA).

## Results

### Systemic administration of high-dose EPO penetrates the BBB and is detectable in brain

#### EPO was measured in CSF in rats

**Figure 1:**
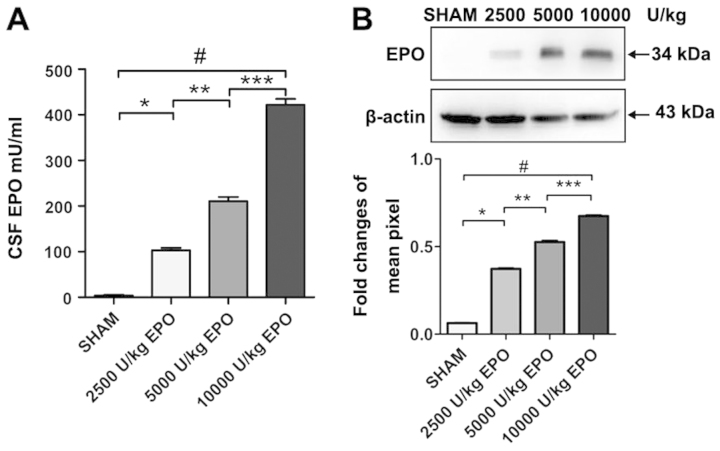
Comparison of cerebrospinal fluid (CSF) erythropoietin (EPO) concentrations in rats after three doses of EPO. (A) Enzyme-linked immunosorbent assay (ELISA) analysis of CSF EPO concentrations. (B) Western blot analysis of CSF EPO concentrations. The EPO levels observed in the CSF depend on the systemically administered doses of EPO. The first group (sham) received an injection of saline, with a CSF EPO concentration at 2.20±1.92 mU/ml. The second group received EPO (2,500 U/kg), with a CSF EPO concentration at 102.61±5.77 mU/ml. The third group received EPO (5,000 U/kg), with a CSF EPO concentration ay 208.62±8.32 mU/ml. The fourth group received EPO (10,000 U/kg), with the CSF EPO concentration at 416.59±9.34 mU/ml. Data are the mean ± SEM; n=7 for each group. ^*^P<0.01 vs. sham; ^**^P<0.01 vs. 2,500 U/kg group; ^***^P<0.01 vs. 5,000 U/kg group; ^#^P<0.01 vs. sham (ANOVA).

As expected, we found a dose-dependent increase of EPO concentration in CSF when comparing 2,500; 5,000 and 10,000 U/kg EPO administered i.p. EPO was undetectable in CSF prior to injection in groups 1–4. Three hours after the EPO injection, however, EPO was detected in the CSF of animals administered 2,500; 5,000 and 10,000 U/kg. Western blot analysis revealed a significant increase in EPO levels in the CSF in the 10,000 U/kg group compared with the 2,500 and 5,000 U/kg group (P<0.01; Fig. 1).

#### EPO was detected in brain

**Figure 2:**
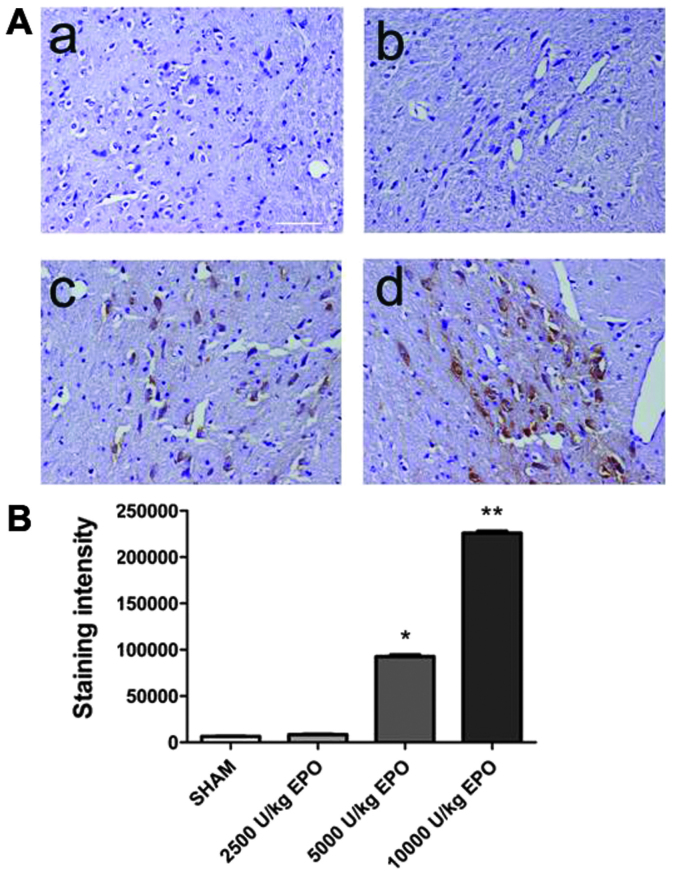
Expression of erythropoietin (EPO) immunoreactivity in brain after three doses of EPO. (A) Immunocytochemistry of EPO in rats’ brain. (B) Statistic of the EPO immunocytochemistry. The first group (sham, a) received an injection of saline. The second group (b) received EPO (2,500 U/kg). The third group (c) received EPO (5,000 U/kg). The fourth group (d) received EPO (10,000 U/kg). Data are the mean ± SEM; n=7 for each group. ^*^P<0.01; ^**^P<0.01 vs. sham (ANOVA). Scale bar, 100 μm.

EPO was undetectable in brain prior to EPO injection. Three hours after the EPO injection, however, EPO was detected in the brain of group 1–4 animals administered 5,000 and 10,000 U/kg. A significant increase in the number of EPO-positive cells in the brain of the 10,000 U/kg group compared with the 2,500 and 5,000 U/kg groups was observed (P<0.01; Fig. 2). A dose of 10,000 U/kg was used in subsequent experiments.

#### EPOR is expressed in the brain

**Figure 3:**
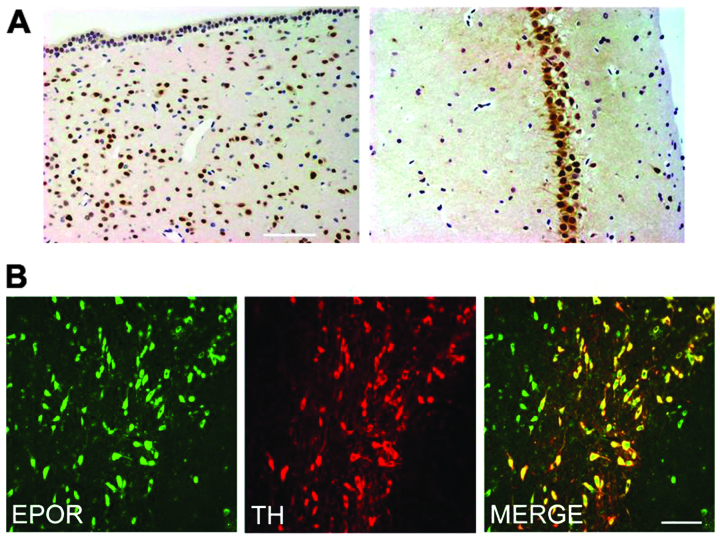
Immunohistochemical analysis of erythropoietin receptor (EPOR) expression in brain sections from rat. (A) Staining of the periventricular zone and hippocampus of rat showed neuron staining for EPOR. (B) Double-staining for tyrosine hydroxylase (TH) showed that TH-immunoreactivity (TH-IR) dopaminergic neurons in the SNpc also express EPOR. Scale bar, 100 μm.

We first determined the expression of EPOR in the brain of rat. The periventricular zone and hippocampus exhibited intense immunoreactivity for EPOR in many medium to large neurons (Fig. 3A). In the SN pars compacta (SNpc), we examined the co-expression of EPOR in TH-IR dopaminergic neurons. Notably, the SNpc TH-IR neurons were strongly immunoreactive for EPOR (Fig. 3B).

### EPO improves behavioral performance

#### Apomorphine-induced rotations

**Figure 4:**
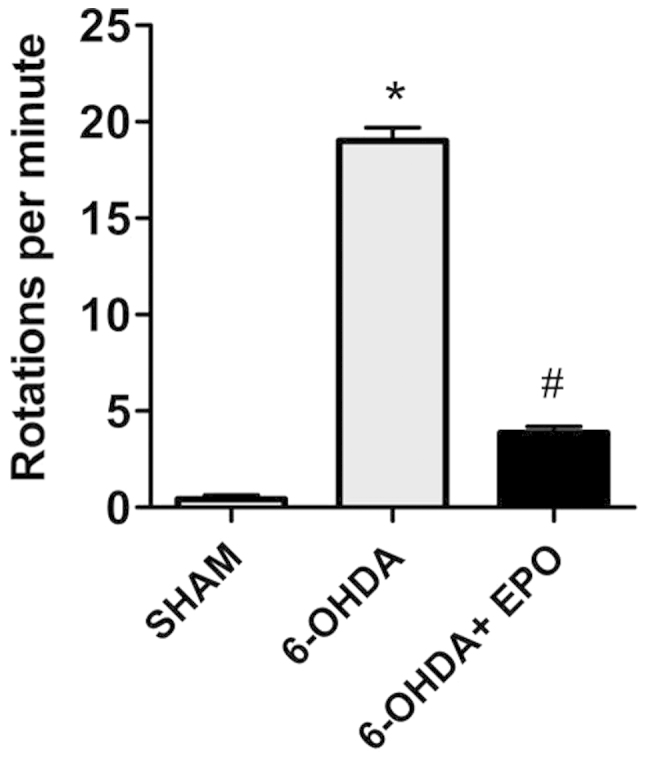
Erythropoietin (EPO) prevents 6-hydroxydopamine (6-OHDA)- induced increases in rotation rates. Data are expressed as the number of net rotations per minute (mean ± SEM) over a 30-min period following apomorphine administration at 21 days after the lesion. ^*^P<0.01 vs. sham-lesion; ^#^P<0.01 vs. 6-OHDA alone, same day (ANOVA); n=7 for each group.

The effect of pretreatment with EPO on 6-OHDA-induced rotational behavior in response to apomorphine was assessed. The rats from groups 5–7 were observed at 7 days prior to the lesion and the mean rotational rate was 0±1, indicating that there was no variability between animals. The apomorphine-induced rotation rates are shown in Fig. 4. The data demonstrate that at 21 days after 6-OHDA administration, rats rotated at mean rates of 19±2 net ipsilateral turns when administered 6-OHDA alone, indicating a lesion of the nigrostriatal pathway. Control (sham-lesioned) rats did not rotate. Intraperitoneal injection of 10,000 U/kg EPO prior to lesion induction was significantly reduced in rotation rates (4±1 turns, respectively; P<0.01; Fig 4).

### EPO decreases dopaminergic neuron loss in the SN

#### TH-positive in the SN

**Figure 5:**
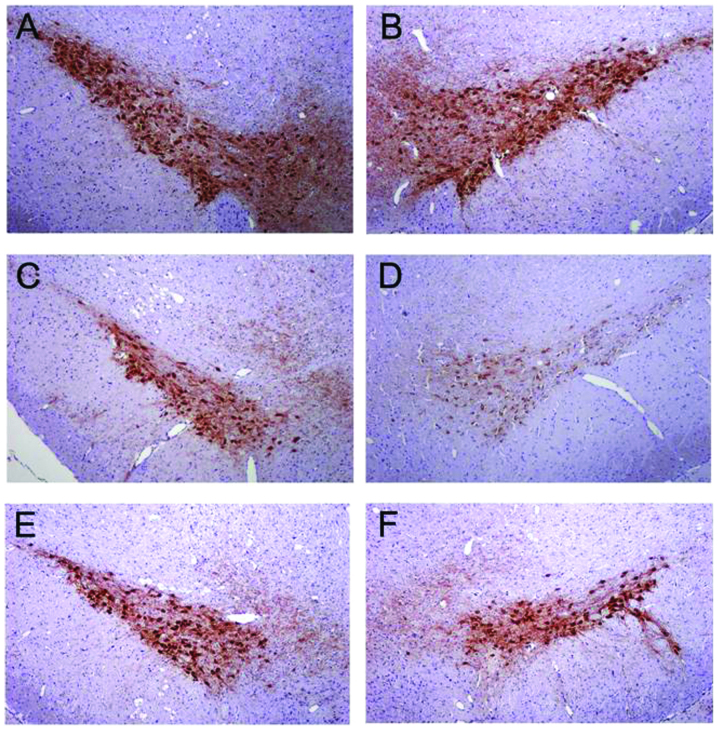
Erythropoietin (EPO) protects 6-hydroxydopamine (6-OHDA)-lesioned dopaminergic neurons. Treatments were as follows: group 5, (A) sham substantia nigra (SN), (B) right SN is lesioned SN; group 6, (C) 6-OHDA SN, (D) right SN is lesioned SN; group 7, rats pretreated with EPO 10,000 U/kg, (E) SN, (F) right SN is lesioned SN; Scale bar, 100 μm.

In control rats receiving saline, the dopaminergic neurons in the SN were intensely immunoreactive to TH (Fig. 5A and B). The number of TH-positive neurons in the injected SN of control rats did not differ from the intact SN of control rats (Fig. 5A and B) or the intact SN of 6-OHDA-lesioned rats and EPO-treated rats (Fig. 5C and E). However, in the lesioned hemisphere of 6-OHDA-lesioned rats, the number of TH-positive neurons in the SN was significantly reduced (Fig. 5D). Numerous TH-positive neurons were found in the ipsilateral SN in the EPO 10,000 U/kg group (Fig. 5F).

#### TH-positive neuron counts in the SN

**Figure 6:**
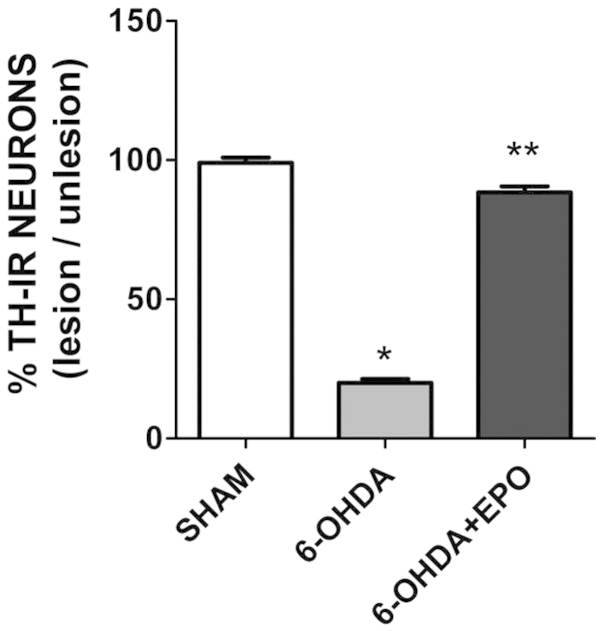
Erythropoietin (EPO) protects 6-hydroxydopamine(6-OHDA)-lesioned dopaminergic neurons. Tyrosine hydroxylase (TH)-positive neurons in the substantia nigra (SN) at 21 days after the lesion. Data are expressed as numbers (mean ± SEM) in the lesioned SN as a percentage of those in the intact SN. ^*^P<0.01 vs. sham-lesion; ^**^P<0.01 vs. 6-OHDA alone; n=5 for each group.

The 6-OHDA lesion induced a significant loss of TH-positive neurons in the ipsilateral SN at 21 days (P<0.01) post-lesion when compared with rats that underwent sham surgery (Fig. 6). There were a number of TH-positive neurons in the lesioned SN in the EPO group 21 days after the 6-OHDA lesion (Fig. 6). In the EPO group, the number of TH-positive neurons in the lesioned SN was significantly increased compared to 6-OHDA (one-factor ANOVA, P<0.01). No difference was found for the TH-positive neurons in the unlesioned SN for each group.

### EPO treatment increased TH levels in the SN and striatum

**Figure 7:**
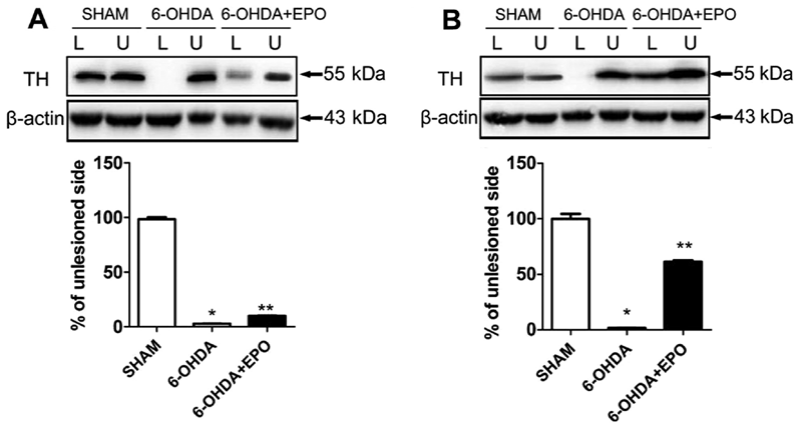
Erythropoietin (EPO) treatment increased tyrosine hydroxylase (TH) levels in the substantia nigra (SN) and striatum. Western blot analysis for TH of extracts from (A) SN and (B) striatum of sham, 6-hydroxydopamine (6-OHDA)-lesioned and 6-OHDA-lesioned plus EPO treated rats. The optical density quantified by densitometry and the value of the lesioned side was expressed as a percentage of unlesioned striatum (lesioned/unlesioned × 100% ± SEM). L, lesioned striatum; U, unlesioned striatum. ^*^P<0.01 vs. sham; ^**^P<0.01 vs. 6-OHDA.

For each rat, the lesioned and unlesioned striatum was studied concomitantly on the same gel, and the results were expressed as a ratio of the lesioned to the unlesioned side. TH proteins in SN, extracted as described in the experimental procedure, were investigated. In 6-OHDA-lesioned rats, the abundance of TH was reduced to 2.4±0.4% in the lesioned SN (P<0.01, compared with sham rats) relative to the unlesioned SN. EPO treatment increased TH levels in the SN, and the abundance of TH was slightly increased to 9.9±0.9% (P<0.01, compared with 6-OHDA-lesioned rats) in the lesioned SN (Fig. 7A). However, an analysis of TH present in striatum revealed marked alterations. In the EPO treatment of lesioned rats, the abundance of TH was significantly increased to 61.5±2.7% (P<0.01, compared with 6-OHDA-lesioned rats) in the lesioned striatum (Fig. 7B).

### EPO suppresses 6-OHDA-induced activation of MAPK pathways

**Figure 8:**
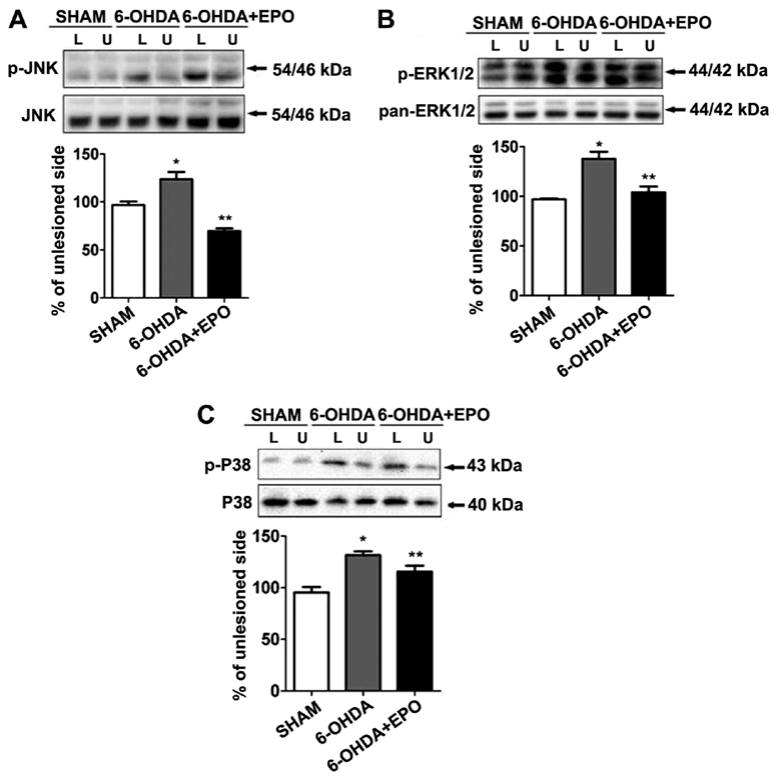
Erythropoietin (EPO) inhibits 6-hydroxydopamine (6-OHDA)-induced activation of c-Jun N-terminal kinases (JNK), extracellular signal-regulated kinase (ERK) and p38. Western blot analysis for (A) JNK, (B) ERK and (C) p38 of extracts from striatum of sham, 6-OHDA-lesioned and 6-OHDA-lesioned plus EPO treated rats. The optical density quantified by densitometry and the value of lesioned side was expressed as percent of unlesioned striatum (lesioned/unlesioned × 100% ± SEM). L, lesioned striatum; U, unlesioned striatum. The inhibition of 6-OHDA-induced JNK, ERK and p38 activation by EPO was shown. ^*^P<0.01 vs. sham; ^**^P<0.01 vs. 6-OHDA.

MAPKs are a specific class of serine/threonine kinases that respond to extracellular signals such as growth factors, mitogens and cellular stress, and mediate proliferation, differentiation, and cell survival in mammalian cells. Four distinct groups of MAPKs exist within mammalian cells: the ERKs, the JNKs, the atypical MAPKs (ERK3, ERK5 and ERK8), and the p38 MAPKs. Since activated MAPK family members, including ERK, JNK, p38, potentially play a role in inflammation and apoptosis, we examined the phosphorylation of MAPKs by western blot analysis using anti-phosphorylated antibodies. As shown in Fig. 8, treatment with 6-OHDA resulted in the robust phosphorylation of JNK, ERK1/2 and p38. Quantification revealed that the levels of phosphorylation were significantly increased for JNK, ERK1/2 and p38 when compared with the sham (P<0.01) (Fig. 8A–C). By contrast, the total amount of JNK, ERK and p38 was not altered during 6-OHDA treatment. We also examined the role of EPO on 6-OHDA-mediated MAPK phosphorylation. Although 6-OHDA treatment elevated levels of MAPK phosphorylation in rats, EPO treatment significantly suppressed 6-OHDA-induced MAPK phosphorylation when compared with 6-OHDA-treated rats (P<0.01) (Fig. 8A–C).

### EPO suppresses 6-OHDA-induced activation of caspase-3

**Figure 9:**
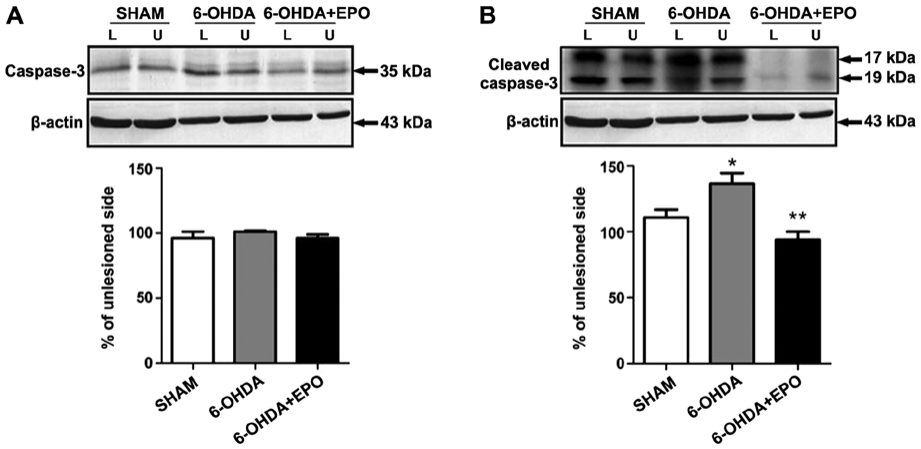
Erythropoietin (EPO) inhibits 6-hydroxydopamine (6-OHDA)-induced activation of caspase-3. Western blot analysis for caspease-3 of extracts from striatum of sham, 6-OHDA-lesioned and 6-OHDA-lesioned plus EPO treated rats. The optical density quantified by densitometry and the value of lesioned side was expressed as percent of unlesioned striatum (lesioned/unlesioned × 100% ± SEM). L, lesioned striatum; U, unlesioned striatum. The inhibition of 6-OHDA-induced caspase-3 activation by EPO was shown. ^*^P<0.01 vs. sham; ^**^P<0.01 vs. 6-OHDA.

Western blot analysis revealed that the activated cleavage product of caspase-3 increased in 6-OHDA treatment as compared to the control. However, pretreatment with EPO significantly suppressed caspase-3 activation (Fig. 9). The effect of EPO on 6-OHDA-induced apoptosis may be, at least in part, mediated by regulating the caspase-3 activation.

## Discussion

Results of the present study have demonstrated that a dose-dependent increase of EPO concentration in CSF as systemic administration of different doses of EPO for rat via i.p. injection, and systemic administration of high-dose EPO exerted neuroprotective effects on a PD model of rats behaviorally and immunohistochemically. A dose-dependent increase of EPO concentration in CSF when different doses of EPO were administerd was found. Systemic administration of EPO prior to injection of 6-OHDA decreased the degeneration of TH-positive neurons in the lesioned SN. An increase of the TH-positive neurons by EPO leads to the improvement of apomorphine-induced rotational behavior. Moreover, EPO reduces the activation of MAPK pathways in the SN and striatum, indicating that an anti-apoptotic effect may be a mechanism of EPO neuroprotection.

The earliest description of EPO in the CNS was that of Tan *et al* (19) and Marti *et al* (20) who demonstrated EPO gene expression in human, monkey and murine brain. In an immunohistochemical study, Juul *et al* (21) found that EPOR and EPO were detected broadly in the neuron in postnatal brains. In addition to neurons, EPO-R is expressed in astrocytes and microglia (22). EPO-R brain expression has been observed during development and adulthood in non-human primates and humans (23). Direct binding of I125-labeled EPO localized EPO binding sites to specific adult brain regions including the hippocampus, cortex and midbrain in mouse. EPO-R expression in adult brain was detected in human in analogous regions and in monkey (24).

The neuroprotective effects of EPO on 6-OHDA-treated dopaminergic neurons were previously examined (12,25,26). No systemic administration of EPO was found in those studies. By contrast, Zhang *et al* (27) used intrastriatal injection of EPO. EPO (80 IU/kg) was administered at 30 min prior to the 6-OHDA lesion. In the present study, 80 IU/kg of EPO was also intrastriatally injected prior to the 6-OHDA lesion. In a previous study, 100 IU/day of EPO was daily administered intraventricularly for 7 days following 6-OHDA lesion (28). In those studies, strong neuroprotective effects of EPO were demonstrated.

The molecular weight of EPO (30.4 kDa) is greater than the molecular weight threshold for lipid-mediated transport across the BBB. Whether EPO is able to cross the BBB is of significance due to implications for its use as a neuroprotective agent. Findings of previous studies have shown that, similar to other large molecule drugs, EPO does not cross the BBB in the absence of BBB disruption (29). Intravenous administration of biotinylated-EPO provided evidence for biotin localization around capillaries in murine brain 5 h after treatment that was eliminated by an increase of unlabeled exogenous EPO, suggesting a specific receptor-mediated transport of EPO into the brain. A pharmacokinetic study directly measured EPO in homogenized brain tissue obtained from neonatal rats and confirmed that systemic EPO is only detected in brain after injection of EPO (5,000 U/kg) and systemic EPO crossed the BBB in a dose-dependent manner, peaking in brain at 10 h (30). The EPO concentrations in the CSF increased after a period of slow equilibration. An increase in the CSF concentration was observed as early as 3 h after intravenous administration. Peak levels were reached between 9 and 24 h (31). In the present study, we found a dose-dependent increase of EPO concentration in CSF. Three hours after EPO injection, EPO was detected in the CSF of rats. Western blot analysis revealed a significant increase in EPO levels in the CSF of the 10,000 U/kg group compared with those of the 2,500 and 5,000 U/kg groups. The dose was determined by a previous study, and 10,000 U/kg of EPO was administered i.p. on a daily basis for 7 days prior to the 6-OHDA lesion. TH-positive fibers were dominantly preserved in the SNc, suggesting that EPO prevented the neurodegeneration of dopaminergic neurons induced by the 6-OHDA lesion.

As previously described, we found that the EPO receptor is co-localized with all TH-positive neurons in the rat SN. It has been demonstrated that EPO can be taken up by neurons. EPO elicits effects on nigral TH expression of doparminergic neurons following 6-OHDA lesion and may prevent neuronal cell death. The mechanism of action of EPO on the nigral-striatal tract may be associated with the property of EPO to reduce neuronal apoptosis (32). In addition, EPO has an antioxidant effect in brain (33), which may ameliorate the oxidative stress in the nigra-striatal tract that is caused by the injection of the 6-OHDA neurotoxin (34). We found the abundance of TH was significantly increased in the lesioned striatum. TH is an enzyme that is responsible for a critical step in the synthesis of dopamine, which occurs in a wide variety of different tissues serving different functions. A possible hypothesis of the TH expression in striatal neurons is that it could rapidly alter in dopamine content. The appearance of TH-positive neurones in the striatum produced by EPO may be a consequence of the increase in striatal dopamine levels. The behavioral performance of rats was also improved.

EPO has trophic effects on dopaminergic neurons. *In vitro* evidence has established that EPO promotes the growth, differentiation, and function of cultured dopaminergic cells (35). EPO also stimulates striatal dopamine release (36). A fundamental mechanism of EPO-induced neuroprotection in cultured neurons is its ability to inhibit apoptosis, reducing both DNA damage and cell membrane asymmetry (37). EPO acts by binding to the EPO-R homodimer. EPO binding activates the phosphorylation activity of JAK2 and phosphorylation of JAK2, which results in signal transduction involving STAT5, PI3 kinase, MAPK and other signaling molecules (38). In a model study, EPOR signaling resulted in strong activation of STAT5 and PI3 kinase/AKT, which were required for neuroprotection, as well as MAPK/ERK1/2 (39).

MAPK pathways play a key role in cell death and survival. MAPKs are a specific class of serine/threonine kinases that respond to extracellular signals such as growth factors, mitogens, and cellular stress and mediate proliferation, differentiation, and cell survival in mammalian cells. We observed that 6-OHDA treatment increased the phosphorylation of all three MAPK members including ERK1/2, JNK, and p38 in a PD rat model as previously described (40). ERK signaling is generally considered a pro-survival pathway (41). However, activation of ERK also contributes to cell death (42). JNK is a major signaling pathway that is activated by oxidative stress and is considered an essential molecule in neuronal cell death. Increased JNK activity has also been reported in the 6-OHDA model (43). JNK-deficient rats exhibited resistance to 6-OHDA- or MPTP-induced injury (44). p38 is known to contribute to the inflammation process as it has been observed *in vivo* (45). Results of that study have also shown that EPO decreased 6-OHDA toxicity by reducing the activation of MAPK pathways thereby protecting the neuron. Many signaling pathways convey apoptotic stimuli in cells. Stress stimulation activates MAPK and various intracellular target proteins, leading to apoptosis. Caspase-3 activation leads to DNA breakage, nuclear chromatin condensation and cell apoptosis. In this study, we found that EPO prevented caspase-3 activation induced by 6-OHDA. Findings of previous studies have demonstrated that EPO can cross the BBB (46) and the systemic administration of EPO improved neurological function of a rat model of stroke. By contrast, systemic injection of EPO at a dose of 5,000 U/kg (body weight) did not protect dopaminergic neurons from 6-OHDA toxicity. Although in this study a dose of 5,000 U/kg was examined a dose-dependent study to determine which dose was effective in the brain of a parkinsonian rat was not conducted (12). Therefore, it cannot be determined whether a higher dose of EPO could protect dopaminergic neurons from 6-O HDA toxicity. In the present study, we performed a dose-dependent study and found that i.p. injection of EPO at a dose of 10,000 U/kg decreased dopaminergic neuron loss from 6-OHDA toxicity in parkinsonian rat. Although the precise mechanisms responsible for EPO need to be determined, the present results suggest that EPO may be neuroprotective via the anti-MAPK pathway.

In conclusion, the systemic high dose of EPO exerted neuroprotective effects by reversing behavioral deficits associated with PD and prevented loss of the dopaminergic neurons through the MAPK pathway. As EPO is used in the clinic, recent pharmacological developments have enabled us to take advantage of EPO without hematopoietic side-effects. Consequently, EPO serves as a potential therapeutic candidate for PD patients.
